# Characterizing the TICAM1 interaction network to target the innate immune response in AD

**DOI:** 10.1002/alz70859_101233

**Published:** 2025-12-25

**Authors:** Hyemin Lee, Elizabeth L Zoeller, Gregory A. Cary, Andrey A Ivanov, Yuhong Du, Allan I. Levey, Haian Fu, The Emory‐Sage‐SGC‐Jax TREAT‐AD Center

**Affiliations:** ^1^ Emory University, Atlanta, GA USA; ^2^ The Jackson Laboratory, Bar Harbor, ME USA; ^3^ Emory University School of Medicine, Atlanta, GA USA

## Abstract

**Background:**

TICAM1 (the TIR domain containing adaptor molecule 1) is part of a risk‐enriched group of proteins related to innate immune response identified through integrated assessment of AD risk considering both genetic association and multi‐omic data. TICAM1 is an adaptor protein that connects activated toll‐like receptors (TLRs) 3 and 4 to the type 1 interferon response as a part of the innate immune system, a key contributor to the development of AD. TICAM1 is upregulated in post‐mortem AD brains, and it has many identified binding partners, providing multiple opportunities for pharmacological intervention. In this study, we validated multiple interactors with TICAM1 and developed screening strategies to target these interactions.

**Methods:**

Meta analysis of existing data covering low and high throughput studies of TICAM1 protein‐protein interactions (PPIs) identified a broad network of proteins annotated as TICAM1 interactors. We experimentally validated these interactions and developed assays to monitor these interactions in screening protocols. As part of a dual validation strategy, we employed both NanoPCA and TR‐FRET assays to characterize the interaction of TICAM1 and thirteen reported protein interactors.

**Results:**

Several reported protein‐protein interactions within the TICAM1 associated network were confirmed by either the NanoPCA or TR‐FRET assays. In combination with our experimental analysis and literature review, the PPIs of TICAM1 with TBK1, TRAF3, and DHX36 emerged as potential opportunities for targeting the TICAM1 pathway by chemical intervention.

**Conclusions:**

Targeting the TICAM1 protein‐protein interactions presents a novel method for regulating the innate immune response. We have identified key PPI and developed screening assays suitable for screening small molecule inhibitors as a first step in drug development addressing the innate immune response in AD. This work has been collected as part of the Emory‐Sage‐SGC‐Jax TREAT‐AD Center.

